# Metabolomic Variability of Different Genotypes of Cashew by LC-Ms and Correlation with Near-Infrared Spectroscopy as a Tool for Fast Phenotyping

**DOI:** 10.3390/metabo9060121

**Published:** 2019-06-25

**Authors:** Elenilson Alves Filho, Lorena Mara Silva, Ynayara Lima, Paulo Ribeiro, Ebenézer Silva, Guilherme Zocolo, Kirley Canuto, Selene Morais, Ana Cecília Castro, Edy de Brito

**Affiliations:** 1 Department of Food Engineering, Universidade Federal do Ceará, Fortaleza-CE 60020-181, Brazil; elenilson.godoy@yahoo.com.br; 2 Embrapa Agroindústria Tropical, Rua Dra Sara Mesquita, 2270, Pici, Fortaleza-CE 60511-110, Brazil; lorena.mara@embrapa.br (L.M.S.); paulo.riceli@embrapa.br (P.R.); ebenezer.silva@embrapa.br (E.S.); guilherme.zocolo@embrapa.br (G.Z.); kirley.canuto@embrapa.br (K.C.); cecilia.castro@embrapa.br (A.C.C.); 3 Centro de Ciências e Tecnologia, Universidade Estadual do Ceará, Fortaleza-CE 60020-181, Brazil; yna.colares@gmail.com (Y.L.); selenemaiademorais@gmail.com (S.M.)

**Keywords:** Anacardium occidentale, fast phenotyping, NIR, UPLC-HRMS, chemometrics

## Abstract

The objective of the present work was to develop an advanced fast phenotyping tool to explore the cashew apple compositions from different genotypes, based on a portable near-infrared (MicroNIR) spectroscopy. This will be in addition to associating the variability of the respective cashew apple pulps with the genotypes by ultra-performance liquid chromatography (UPLC), coupled with high-resolution mass spectrometry (HRMS). The NIR analysis is a non-destructive, low-cost procedure that provides prompt results, while considering the morphology of different cashew apples (shape, size, and color). The UPLC-HRMS analysis is characterized by specific bioactive compounds, such as the derivatives of hydroxybutanoic acid, galloyl, and flavonoids. Furthermore, both techniques allowed the identification of a group of accessions, which presented similarities among the chemical profiling. However, to improve the understanding of cashew chemical and physical variability, further variables related to the cashew apple composition, such as edaphoclimatic conditions, should be considered for future studies. These approaches lead to the conclusion that these two tools are useful for the maintenance of BAG-Caju (Cashew Germplasm Bank) and for the cashew-breeding program.

## 1. Introduction

Cashew (*Anacardium occidentale* L.) is a fruit tree originating from the north of South America, with greater dispersion in the coastal regions, from the state of Rio de Janeiro to the Amazon, where it is possible to find spontaneous populations with great genetic and, consequently, phenotypic variability. To conserve part of this variability, Embrapa maintains a germplasm bank (BAG-Caju) holding almost seven hundred accessions with important variability [1].

Nuts are true fruits with hard shells that are inedible due to the presence of cardol and cardanol (caustic substances). The cashew nut consists of nut kernels that are edible and commercially known as cashew nuts. This complete fruit forms on the distal end of the false fruit that is also edible (called cashew apple or peduncle) [2]. Cashew nut (kernel) is the main product of this crop marketed globally. However, Brazil, by tradition and culture, has the habit of consuming the cashew apple (false fruit) in different ways, e.g., as fresh fruit, juice, or sweets. Thus, BAG-Caju that was initially established based on the size and weight of the cashew nut began to include the variability related to the false fruit, such as vitamin C, sugars, acidity, astringency, color, and bioactive compounds [3].

Due to the elevated number of cashew apple accessions, we have investigated the applicable potential of various technologies to develop simple, precise, and low-cost methods for studying the physico-chemical and nutritional features of this fruit. Among the technologies, the near infrared (NIR) spectroscopy is considered to be an economical, fast, and efficient technique for the evaluation of foodstuffs [4,5,6]. The NIR analysis produces an electromagnetic spectrum (reflectance or transmittance) between 780 nm and 2,500 nm, where the wavelength depends on the scattering and absorption processes according to the chemical compositions: molecular functional groups, C-H, N-H, S-H, or O-H bonds. A MicroNIR spectrometer is a portable NIR device for real-time, in situ, and non-destructive chemical and physical analyses [7].

In particular, the cashew apple exhibits beneficial characteristics to human health, such as antitumor, antimicrobial, urease inhibitory, and lipoxygenase activities [3,8,9,10]. The genetic enhancement of the quality and attributes of the cashew apple is still under development in Brazil, although the knowledge of genetic diversity for the construction of germplasm banks exists. Most cashew breeding programs are based on traditional selection approaches, including the size and weight of nuts or yield of a cashew tree, and efforts have also been applied in the prospection of dwarf genotypes with enhanced fruit quality and resistance to pests and diseases [11]. Therefore, the identification of the characteristics with economic interest, related to conserved or cultivated accessions, may be appropriated by breeding programs aimed at launching high-performance cultivars to meet specific demands, such as cultivars with high levels of bioactive compounds. The analysis of unique chemical fingerprints, based on ultra-performance liquid chromatography (UPLC), coupled with high-resolution mass spectrometry (HRMS) has been successfully applied for the study of fruit pulps. In such foodstuff studies, a large number of samples have to be screened for identifying metabolites and their classification according to the geographic origin, climate conditions, genotype, and cultural practices using multivariate statistics [3,12,13,14,15]. Usually, multivariate analyses are applied, in order to explore complex matrices, such as foodstuff to determine the variations and relationships among the compositions of the samples [16,17]. This untargeted approach is advantageous when the compounds and degradation products are not known.

From the foregoing studies, the aim of the present study was to evaluate the potential of a MicroNIR spectrometer to explore the composition of different genotypes of intact cashew apple from the Embrapa germplasm bank, as well as to create multivariate regression models considering °Brix, total acidity, and concentrations of ascorbic acid (vitamin C). Furthermore, due to the need to investigate plants that produce fruits with numerous compounds beneficial to human health, the study of metabolite profiling (nutritional and functional compounds) to reveal the variability of pulps from different genotypes of cashew apple was also investigated.

## 2. Results

The results were divided according to the analytical technique applied to evaluate the cashew apple variability: Section 2.1 for MicroNIR and Section 2.2 for UPLC-HRMS.

### 2.1. Exploratory Multivariate Analysis of the MicroNIR Dataset

The NIR analysis contains undesirable sources of error (intrinsic imperfections) for multivariate studies, and therefore, pre-treatments can correct many systematic or random errors. The information from the cashew apple composition was partly obscured (overlapped), and the distribution of the variables was highly skewed by usual and expected spectral imperfections. Three pre-treatments of the MicroNIR dataset were tested to assess the features regarding the cashew apple composition, minimizing irrelevant variations within the spectra to obtain quality data: Multiplicative scatter correction (MSC), standard normal variate (SNV), and first derivatives, using Savitzky–Golay filter with a second order polynomial for five points (Figure S1 in Supporting Information). Therefore, the pre-processing by MSC was chosen as an input for the following chemometric analysis.

**Figure 1:**
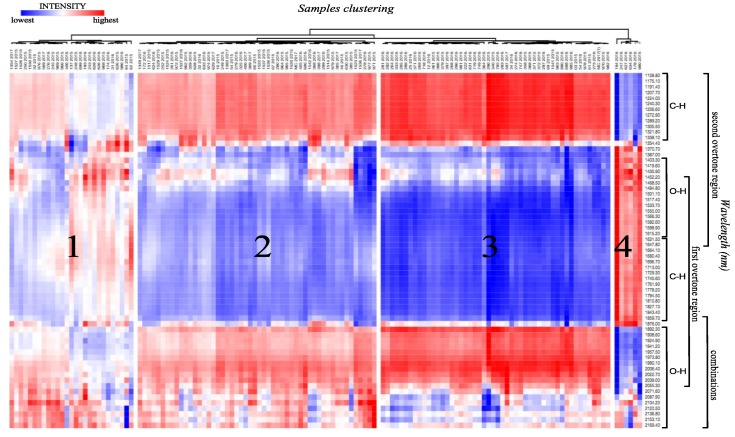
Three-dimensional (3D) dendrogram (sample × wavelength from MicroNIR × intensity) representing the chemical composition similarity relationships among the genotypes.

To comprehend and classify the variability of the organic compounds in intact cashew apples according to the genotype, via detecting the tendencies related to the composition, a method based on hierarchical clustering was developed to segregate the samples in groups according to the composition similarity. Figure 1 presents a 3D dendrogram in heat map form: Genotypes with the harvest year in columns; wavelength in rows, and the signals intensities illustrated in colors. The relatively deep red represents the high relative intensity (wavelength); the relatively deep blue, the low relative intensity; and white, the intermediate intensity. Important tendencies for four cluster formations were observed in the heat map based on genotype dissimilarity (Figure S3 in Supporting Information). Cluster 4 presented the most dissimilar genotypes (zero similarity). The results reflected the natural differences among the groups, which were formed by different spectroscopic information from cashew genotypes pulp composition.

#### Multivariate Regression Analysis of the MicroNIR Dataset

**Figure 2:**
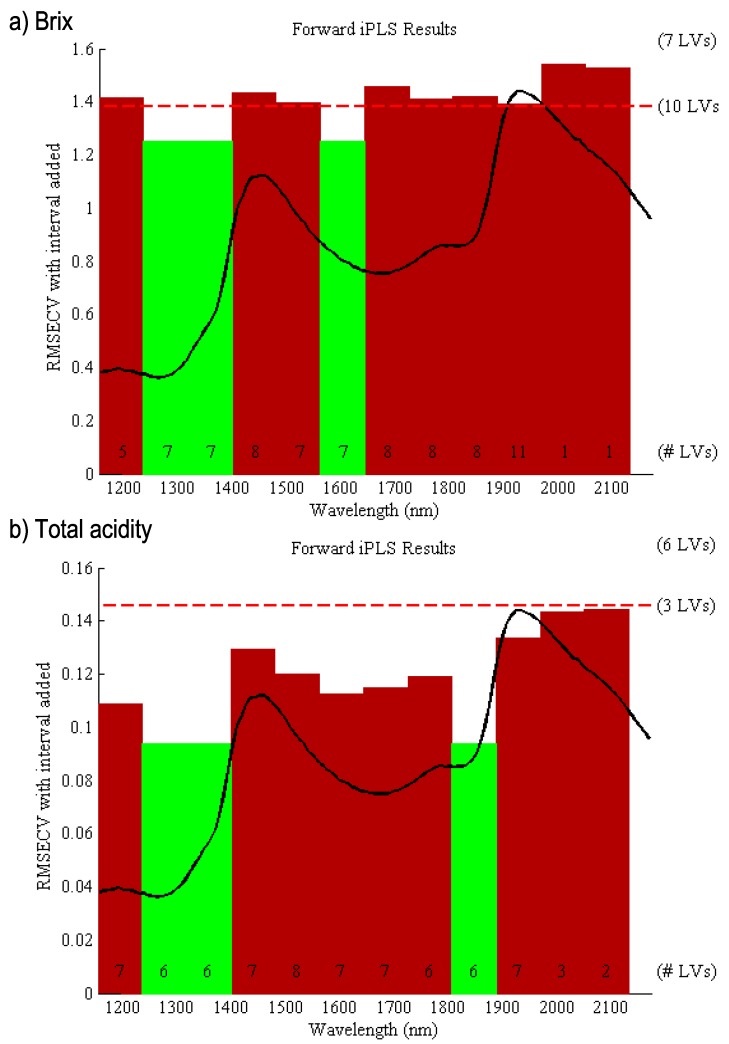
Spectral average (black line) of the MicroNIR spectra from different genotypes of cashew. The relevant absorbance selected by the iPLS model (green) for the genotype discrimination based on (**a**) °Brix and (**b**) total acidity.

Prior to performing the partial least squares (PLS) analysis, the °Brix value, total acidity, and concentrations of ascorbic acid (vitamin C) were determined in 31 cashew apples randomly chosen from the set of accessions. Three regression models were created for each dependent variable (°Brix, total acidity, and ascorbic acid) with spectroscopic data to evaluate the association between the chemical variability, genotype and NIR profile. Sequential to these supervised regression modeling, the PLS by intervals, known as iPLS (interval PLS), was developed to maximize the covariance between the independent variables on the MicroNIR dataset (X matrix) and each dependent variable. This method realizes individuals PLS models for each pre-defined spectra intervals, optimizing the predictive capacity of the model, while assisting the interpretation by reducing the number of variables, thereby providing superior prediction capacity using all the variables [18]. Figure 2 illustrates the relevant intervals (highlighted in green color) for regression modeling, based on RMSECV using the Brix values (a) and total acidities (b). The modeling using the concentrations of ascorbic acid was weakly adjusted based on statistical parameters (data not shown).

The merit graph obtained by regression modeling, using °Brix and total acidities, were evaluated to assess the quality of the calibration models, which are illustrated in Supplementary Information (Figures S4 and S5). The Hotelling’s T^2^ × Q residuals graph indicates that the sample did not negatively influence the modeling [19]. The leverage plot revealed the influence of each genotype on models based on Hotelling’s T^2^, and the studentized residuals (mean zero and unit variance) indicated the lack of fit of some quantitative parameters [20]. However, despite the high leverage and high residuals, the respective genotypes expressed a very low studentized Y residual, and therefore, the regression model was still able to sufficiently predict the °Brix and total acidity based on selected MicroNIR spectra (green region in Figure 2). The robustness of both prediction models (°Brix and total acidity) was achieved by the proximity between both regression curves from calibration and cross-validation (red and green lines). Furthermore, the statistical parameters used to assess the modeling quality (Table 1) indicated a well-adjusted models for both °Brix and total acidity, according to the high total variance cumulated using three latent variables (LVs) (higher than 94%), low bias model, low calibration and cross-validation errors, and proximity between the calibration and cross-validation errors. Additionally, the root mean square error of calibration (RMSEC) and root mean square error of cross-validation (RMSECV) ratio, close to 0.75, is indicative of a well-adjusted model [21,22].

### 2.2. UPLC-HRMS

Commonly, in UPLC-HRMS analysis, some organic compounds may preferentially ionize in the positive or negative ionization mode, as phenolic and carboxylic derivatives ionized well in negative ionization mode, while flavonoids and alkaloids ionize better in positive ionization mode. Therefore, the cashew apple pulps were analyzed under a negative ionization mode to screen the aforementioned compounds and differentiate each cashew apple pulp according to the genotypes. Due to the complexity of the chromatographic data, visual differentiation of the sample composition could not be achieved. Therefore, non-targeted multivariate analyses by hierarchical cluster analysis (HCA) and principal component analysis (PCA) were applied to comprehend the variability of the secondary metabolites in cashew apple pulps according to the genotype.

**Figure 3:**
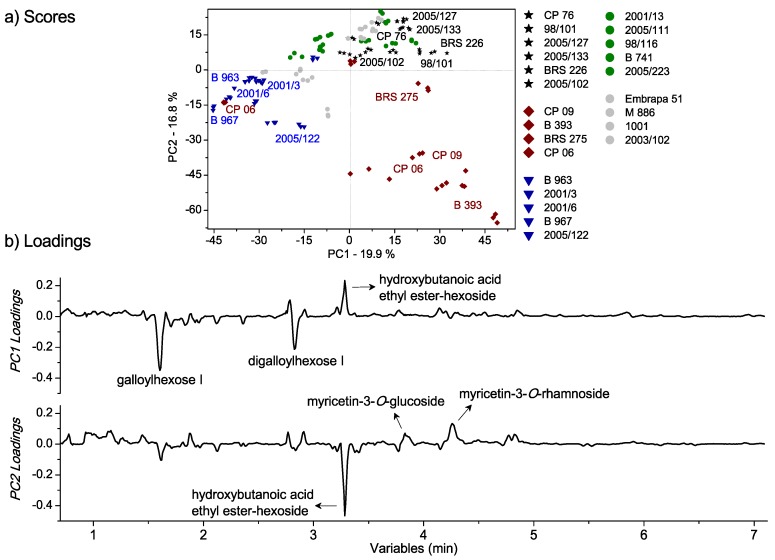
Principal component analysis (PCA) multivariate analysis of UPLC-HRMS data from cashew apple pulps of different genotypes: a) PC1 × PC2 scores coordinate system for the cashew apple pulps from different genotypes; b) respective loadings plotted in lines form. The samples were assigned symbols according to the clustering tendency: blue triangles for negative values of PC1 and PC2; red squares for positive values of PC1 and negative values of PC2; black stars for positive values of PC1 and PC2; green circles for positive values of PC2. The samples that did not present relevant results were symbolized by gray circles.

Initially, the unsupervised method HCA was applied: Segregating the pulps in groups according to similarity. Important tendencies for four cluster formations were observed in the dendrogram based on the genotype at a similarity index of 0.362 (Figure S3 in Supporting information). Cluster 4 presented the most distant samples included in the study, with zero similarity (as also observed in Figure 1). The results reflected the natural differences among the groups, which were formed as a function of pulp composition, dependent on the cashew genotype. In addition, the PCA method was applied to assist the modeling and interpretation of the multivariate data, with the scores graph presented in Figure 3a, and the respective loadings (plotted in lines) in Figure 3b. The tentatively identified biomarkers are presented in Table S1 (Supporting information). The main separation tendencies of the samples were observed with respect to PC1 and PC2 axes with a total explained the variance of 36.7%. The clusters observed in HCA were important for identifying the resultant grouping in the scores graph. The pulps were assigned symbols according to the clustering tendency: Blue triangles for negative scores of PC1 and PC2; red squares for positive scores of PC1 and negative scores of PC2; black stars for positive scores of PC1 and PC2; and green circles for positive scores of PC2. The samples with no relevant result were symbolized by gray circles considering the unrepresentative replicates according to the year.

**Figure 4:**
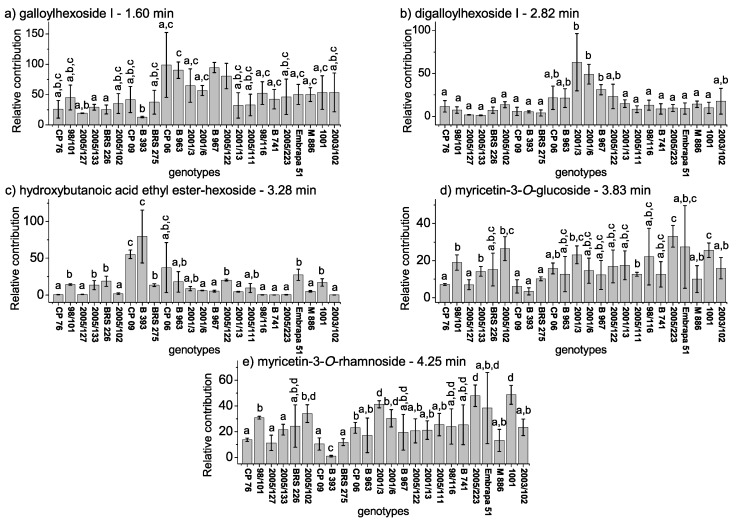
Relative contributions of the isotopic forms in chromatograms (UPLC-HRMS) for the compounds at retention times of 1.60, 2.82, 3.23, 3.82, and 4.25 min.

Compounds with significant changes, based on genotypes according to the chemometric evaluation, and not exhibiting overlapping signals, were integrated (details in Experimental Section 4.2.2), and their variations were expressed as the relative contribution. The relative peaks areas were calculated for quantitative expression of the chemical properties, with the differences evaluated by ANOVA single factor. Figure 4 illustrates the relative contributions of the total ion abundance of the peaks in the pulps, since the relative amplitude of the peaks provided the relative population of the isotopic forms in the chromatograms. The results from the signal area of the base peak intensity (BPI) corroborated the PCA results, considering the deviation of the method from the three replicates of sampling during two years, which totaled 6 pulps for each genotype, with the hydroxybutanoic acid ethyl ester-hexoside (at 3.28 min) being responsible for the pulps clustering at the red group, and galloylhexose I (at 1.60 min) and digalloylhexoside I (at 2.82 min) for the pulps clustering in the blue group.

#### 2.2.1. Multivariate Classification Analysis of the UPLC-HRMS Dataset

**Figure 5:**
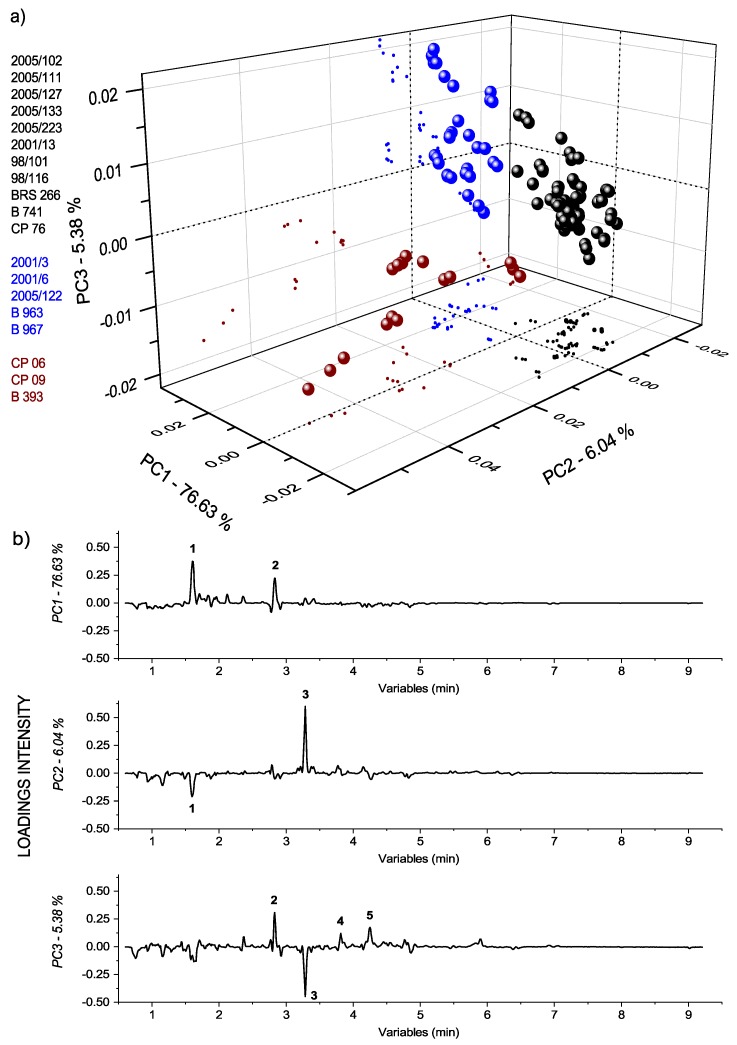
PLS-DA classification model of UPLC-HRMS data from cashew apple pulps of different genotypes: (**a**) LV1 × LV2 × LV3 scores 3D plot; (**b**) respective loadings potted in lines form (b) from PLS-DA for classification of the cashew apple pulps. Legend: 1-galloylhexose I (1.60 min); 2-digalloylhexoside I (2.82 min); 3-hydroxybutanoic acid ethyl ester-hexoside (3.28 min); 4-myricetin-3-*O*-glucoside (3.83 min); and 5-myricetin-3-*O*-rhamnoside (4.25 min).

Based on the non-targeted chemometrics results and the relative quantification, the classification modeling by partial least squares-discriminant analysis (PLS-DA) was employed to improve the association of the chemical variability of the pulps according to the cashew genotype, which is illustrated in Figure 5. The model presented a classification ability of 88.05% using 3 LVs, considering the year replicate. The statistical parameters used to assess the quality of the modeling (Table 2) indicated a well-adjusted classification, with an RMSEC/RMSECV ratio close to 0.75 (similar values) [22]. The low calibration and cross-validation errors expressed a suitable predictive performance of the model estimated as a function of the global error, samples leverage, and the sample residual X-variance. The low error values indicated the similarity between the pulps used for prediction and those used to make the calibration model.

## 3. Discussion

It is known that NIR spectroscopy is a non-destructive, low-cost, and non-invasive procedure providing prompt results for a sample composition: Any molecule containing C-H, N-H, S-H, or O-H bonds. However, due to the intrinsic overlapping of the signals, pre-processing is mandatory. The MSC and SNV algorithms presented similar effects, which were considered as exchangeable (Figure S1 in Supporting Information). After the application of the MSC algorithm, the spectra were adjusted to present the same scatter level estimated by a mean spectrum. Conversely, the SNV algorithm treated each spectrum separately by absorbance autoscaling [23]. The derivative algorithm emphasized steep peaks and enhanced the overlapping peaks, as well as reduced the measurement variations, thereby improving the differentiation of the bands. The first and/or second derivatives are more commonly applied, and the second derivative NIR spectrum may result in sharp signals [24,25]. Therefore, the MSC algorithm was chosen for the development of the experiments.

According to the HCA-heat map (Figure 1), important tendencies for four main cluster formations were observed based on the genotype, even with differences in fruit morphology, such as shape, size, and peel color. Another disadvantage of NIR analysis was the penetration of radiation into the tissues of fruits, which skin may reduce the light penetration that decreases with the depth [26]. The results reflected the natural differences among the cashew apple groups formed as a function of the composition intrinsically related to the genotype. Cluster 4 presented the most dissimilar samples compared to those of the other groups. The main absorbance peaks, related to the clustering, were located between 1150 and 1340 nm, 1370 and 1850 nm, and 1900 and 2020 nm. The absorbance from 2040 to 2170 nm was characterized as indicative of non-relevant functional groups. The absorbance from 1400 to 1620 nm and 1900 to 2020 nm were characterized as the second and first overtone of the OH stretch, respectively. The absorbance from 1150 to 1340 nm and 1650 to 1850 nm were due to the C-H stretches related to the second, and first overtone, respectively, which may be from carbohydrates and other organic compounds present in the cashew apple skin and/or pulp. The band at 1340 nm is attributed to the CH group from cellulose [27]. Additionally, it was highlighted that the O-H group from monomeric organic acids presented the first overtone at 1445 nm; and a characteristic overtone around 1890 nm indicative of the O-H stretching combined with C-O stretching from organic acids [26,28]. However, it is known that all these absorption bands are close to the stronger water absorption regions, hindering the signals observation [29].

The iPLS evaluation highlighted the region between 1250 and 1400 nm as the most important for fruit discrimination, based on the calibration modeling by °Brix values and total acidity (statistical parameters, described in Table 1. The modeling for ascorbic acid was weakly adjusted based on statistical parameters. In particular, the absorbance range between 1570 and 1650 nm was important for the °Brix model, while the absorbance between 1800 and 1900 nm was important for the total acidity. The absorbance between 1650 and 1850 nm may be related to the C-H stretches from the second, and first overtone, respectively (Figure 1), which may be from carbohydrates and other organic species in cashew apple skin and/or pulp; and between 1800–1900 nm may be related to carbonyl and carboxyl groups from carboxylic acids [27]. The regression modeling °Brix values (a) and total acidities (b) were well adjusted based on statistical parameters, and the modeling for ascorbic acid was weakly adjusted. This may be because the MicroNIR was operated between 1150–2170 nm, and some organic acids found in fruits typically show bands from O-H group related to the second and third overtones approximately at 1000, and 800 nm, respectively, as well as starch and sugars to the second (920 nm) and the third (720 nm) overtones of O-H stretching, and the third (910 nm) and the fourth (750 nm) overtones of C-H stretching [28].

The relatively low correlation coefficients (r^2^) for both models (°Brix values and total acidity) express the rather weak calibration performances estimated as a function of the global model error, samples leverage, and the sample residual X-variance. Therefore, the cross-validation results indicated that further parameters related to the fruits morphology (shape, size, and color), non-homogeneous distribution of particles into the fruits (density variations), and environmental factors that may affect the instrument performance as the illumination (since all the analysis was developed during the year), must be taken into account for improving the comprehension of the chemical and physical variability of the cashew apples from the germplasm bank of Embrapa. For instance, small physical variations from sample to sample may lead to light scattering that influences the MicroNIR measurement, resulting in baseline shifts and scaling variations (intensity variations), and consequently, disturbing the future predictions evaluation.

Prior to the chemometric analysis of the HPLC-HRMS analysis, a valuable feature of chromatograms acquisition was taken into account, since the retention times of the chromatographic peaks are sensitive to minor fluctuations in temperature, pH, flow, pump operation, etc. To solve the problem of small peaks shifts related to the same compound inter-chromatograms, some different peak alignment methods were tested. The alignment practice can be performed manually, using COW (correlation optimized warping) [30], or using the bucketing method, which reduces the chromatogram dimensionally by slicing it into equal sized regions [31], making it easier to analyze the respective loadings. Therefore, all the chromatogram peaks were previously aligned using the COW method, which is illustrated in Figure S2 in Supporting Information.

Significant composition variability was detected in PCA regarding cashew genotypes despite the low cumulated variance, which indicated the existence of further factors beyond the scope of this study, such as seasonality. An examination of the loadings provided evidence of the variables (compounds) responsible for the separations or clustering observed in scores. The signals from galloylhexose I (at 1.60 min, 331.0650 *m/z*), digalloylhexoside I (at 2.82 min, 483.0741 *m/z*), hydroxybutanoic acid ethyl ester-hexoside (at 3.28 min, 293.1242 *m/z*), myricetin-3-*O*-glucoside (at 3.83 min, 479.0826 *m/z*), myricetin-3-*O*-rhamnoside (at 4.25 min, 463.0875 *m/z*), and a mixture of unknown compounds (at 4.82 min) were responsible for the pulps placement based on genotypes. Minor compounds were irrelevant due to the pretreatments of mean centering applied over the samples [16]. According to Figure 3, the negative loadings of PC1 are the pulps symbolized by blue triangles (2001/3, 2001/6, 2005/122, B 963, and B967) with relatively high amounts of galloylhexose I and digalloylhexoside I. This latter compound is a trihydroxybenzoic acid derivative, which provides astringent flavor and can contribute to the characteristic bitter taste of immature cashew apple [32]. The pulps symbolized by red squares (CP 06, CP 09, B 393, and BRS 275) and black stars (2005/127, 2005/133, 2005/102, BRS 226, CP 76, 98/101) exhibited tendencies of having relatively high amounts of hydroxybutanoic acid ethyl ester-hexoside at a retention time of 3.28 min according to positive loadings of PC1. Hydroxybutanoic acid ethyl ester-hexoside has been previously described in melon fruit and it is considered a precursor of volatile compounds [33]. The presence of this compound has been associated with some amino acids, such as alanine, glutamine, isoleucine, phenylalanine, tryptophan, and tyrosine. Alanine has been reported as one of the key amino acids of the characteristic profile of cashew apple. Phenylalanine and tyrosine have also been detected but in small amounts [34]. The positive loadings of PC2 exhibited the tendency of the cashew apple pulps symbolized by green circle (2005/111, 2005/223, 2001/13, 98/116, and B 741) and black stars (2005/127, 2005/133, 2005/102, BRS 226, CP 76, 98/101) to have relatively high amounts of the flavonoids, myricetin-3-*O*-glucoside, myricetin-3-*O*-rhamnoside, and an unknown mixture of compounds between the retention times of 4.79 and 4.85 min. The presence of myricetin-derived and other flavonoids compounds in pulps may offer biological benefits, including the reduction of cardiovascular disease and risks of cancer [3], as well as antihyperglycemic property [35]. Furthermore, these compounds have been reported in methanol-water extracts of cashew apple [3]. The corroborative results between quantitative and chemometric analyses confirm the advantages of an untargeted multivariate analysis, since the compounds and degradation products are not always known, and it is sometimes difficult to find certified standards.

According to Figure 5, the LV1 axis was most relevant in clustering the cashew apple pulps in black color (2005/102, 2005/111, 2005/127, 2005/133, 2005/223, 2001/13, 98/101, 98/116, BRS 266, B 741, CP 76), and in separating them from those in red (CP 06, CP 09, B 393) and blue (2001/3, 2001/6, 2005/122, B 963, B 967). In addition, the LV2 axis was important in clustering the red samples at the positive scores, and LV3 was relevant in the separation of the red and blue cashew apple pulps. The interpretation of the loadings revealed that the pulps in black had higher amounts of galloylhexose I and digalloylhexoside I than those of in red and blue. The results corroborated that the pulps in red color have a higher amount of hydroxybutanoic acid ethyl ester-hexoside than those in the black and blue pulps; in addition, opposite behavior between the hydroxybutanoic acid ethyl ester-hexoside and galloylhexose I was presented. Finally, the LV3 axis confirmed the relatively high amount of the flavonoids, myricetin-3-*O*-glucoside and myricetin-3-*O*-rhamnoside, in the pulps in blue and presented the opposite behavior between these flavonoids and hydroxybutanoic acid ethyl ester-hexoside. Therefore, the statistics data indicated that the model could be acceptable to classify new or unknown cashew apple pulps based on the main secondary metabolites.

## 4. Materials and Methods

### 4.1. Sampling and Experimental Planning

Based on experimental viability, 764 cashew apples (*Anacardium occidentale*, L.) from 24 different accessions were randomly harvested at the Embrapa experimental station (Pacajus, Ceará, Brazil)— coordinates 4°11′07”S; 38°30′07”W; altitude: 70 m. The region has a tropical climate, average temperatures of 26 to 28 °C, and 1020 mm average annual rainfall. The soil is classified as Ultisol and has a sandy/medium texture with low organic matter content. The cashew samplings were collected between August and December in two years. Table 3 presents the different genotypes with their respective accession numbers and morphoagronomic characteristics.

### 4.2. Portable NIR Spectrometer Analysis

The portable NIR (MicroNIR) analysis of the cashew apple composition was divided into two stages. First, all the 764 intact fruits were analyzed by MicroNIR. After that, 31 fruits were randomly selected for the determination of quantitative parameters, such as °Brix, total acidity, and concentration of ascorbic acid (vitamin C) to develop multivariate regression models.

The NIR experiment was acquired using a portable NIR spectrometer (MicroNIR 1700, Viavi, Milpitas, CA, USA), which operated in a range between 1150 and 2170 nm (spectral resolution of 10 nm,), with dimensions of 45 mm diameter × 42 mm high, two tungsten sources for reflectance measurements, and a continuous monochromator based on a linear variable filter. The parameters for spectral data acquisition were set at 50 ms integration time and an average of 100 scans. The reference spectrum for the absorbance calculation was obtained from a piece of Spectralon™, and the dark signal was obtained by pointing the measurement window of the instrument to the ambient environment.

#### 4.2.1. Determination of °Brix, Total Acidity, and Concentration of Ascorbic Acid

The °Brix, total acidity, and concentration of ascorbic acid (vitamin C) were determined in 31 cashew apples randomly chosen from the set of accessions. These experiments were carried out to use the quantitative results as categorical variables (Y column) to develop multivariate regression models by partial least square (PLS) analyses by maximizing the covariance between X matrix (NIR spectral data) and Y responses (°Brix, total acidity, and ascorbic acid as dependent variables).

The °Brix (concentration of sucrose w/w) was determined following the AOAC method (2005) [36] (soluble solids content), which was obtained by refractometry using a digital refractometer (ATAGO™ N1, Kirkland-WA-USA) with automatic temperature compensation.

The total acidity was determined as follows: Total titratable acidity (TTA) determined by titration with NaOH solution (0.1 N) in 1 g of the pulp diluted to approximately 50 mL of distilled water, containing 3 drops of 1% phenolphthalein until pink coloration, was observed. The results were expressed as the percentage of malic acid according to IAL (1985) [36].

The concentrations of ascorbic acid were determined by titration with 0.02% 2,6-dichloro-indophenol (DFI) as reported by Strohecker and Henning (1967) [37]. One gram of pulp was diluted to 100 mL with 0.5% oxalic acid and homogenized. Subsequently, 5 mL of this solution was diluted to 50 mL with distilled water and titrated. The results were expressed as mg·100 g^−1^ FW (fresh weight).

#### 4.2.2. Chemometric Analysis of the MicroNIR Dataset

Different multivariate approaches were performed on the numerical matrix from 764 cashew apple fruits (Section 2.1). The averaging method was applied on those fruits from the same accession (from four to six replicates), resulting in 135 mean spectra. The spectral region between 1150 and 2170 nm was used for the modeling, and a matrix with the dimensionality of 16,875 data points (135 spectra × 125 variables into each spectrum) was generated. The samples were named according to the accession number and year of harvest.

For numerical matrix construction, each spectrum was converted to an American Standard Code for Information Interchange (ASCII) file and imported by the Origin™ program (version 9.4). To reduce the dimensionality of the original data and to assist the interpretation of the multivariate dataset, the matrix was averaged along variables by a factor of 2 using the PLS-Toolbox™ program (version 8.6.2, Eigenvector Research Incorporated, Manson, WA USA), and imported by GENE-E program (https://software.broadinstitute.org/GENE-E/index.html) for pattern recognition through the hierarchical clustering algorithm by heat map. The Euclidean distance was used to measure the proximity between the samples (columns), and the average linkage method (sum-of-squares approach in calculating intercluster distances) was applied. The results were presented as heat maps (three-dimensional (3D) dendrogram = sample × wavelength × intensity) [22].

In addition to the unsupervised analysis, supervised methods by PLS were developed using the °Brix values, total acidity, and concentrations of ascorbic acid previously calculated in Section 2.2.1 to improve the identification of chemical changes according to genotypes by MicroNIR. The simplified PLS (SIMPLS) algorithm was applied to build the models and the LVs were selected in accordance with the statistical parameters based on the full cross-validation method: RMSEC, RMSEV, and calibration and cross-validation coefficients (r^2^) [16].

### 4.3. UPLC-HRMS Analysis

Due to the health benefits of the cashew apple, additional experiments were developed in parallel by non-targeted UPLC-HRMS analysis. A total of 24 different genotypes of cashew apple (as described in Section 2.1) were evaluated. The cashew apples were manually pressed to obtain the resultant pulp, followed by centrifuged for 5 min at 804.6 g (IEC clinical centrifuge, Damon/IEC Division, Needham, MA, USA). The samples were preserved at −80 °C until the analysis.

Prior to the UPLC-HRMS analysis, the samples were filtered using PTFE membranes of 0.22 μm. The analysis was performed on an Acquity system (Waters) coupled with quadrupole/TOF (Waters) equipped with an ESI source operated in the positive ion mode. The chromatographic separation was performed using Waters Acquity UPLC BEH (150.0 × 2.1 mm, 1.7 μm) column with the temperature set at 40 °C. Water and acetonitrile were used for the mobile phase, both with 0.1% formic acid. The gradient ranged from 2% to 95% of water in 15 min in a flow of 0.4 mL·min^−1^ and injection volume of 5.0 μL per sample. The desolvation gas was N_2_. The desolvation temperature was set at 350 °C at a flow rate of 350 L·h^−1^ and a source temperature of 120 °C. The capillary voltage was set to 3200 V. The collision energies/cone voltages were set at 6 eV/15 V (low) and 30–50 eV/30 V (high) to achieve sufficient fragmentation. Data were collected using the negative ionization mode between 100 Da and 1180 Da, and the mode tandem was MS^E^.

#### 4.3.1. Chemometric Analysis of the HPLC-HRMS Dataset

Chemometric analysis was performed on the numerical matrix from 24 cashew apples harvested in duplicates this year, and analytical triplicate, resulting in 144 chromatograms. The chromatographic region between 0.65 and 7.12 min was selected. The samples were named according to the accession numbers (Table 3).

The same procedure applied for numerical matrix construction from MicroNIR (Section 4.2.2) dataset was also applied for the UPLC-HRMS dataset. Therefore the chromatograms were converted to an American Standard Code for Information Interchange (ASCII) file and import by the Origin™ program (version 9.4) in order to build the matrix. The final matrix was exported for chemometric analyses by HCA, PCA, and PLS-DA using PLS Toolbox™ program (version 8.6.2, Eigenvector Research Incorporated, Manson, WA, USA).

The normalized scaling parameter and baseline correction, using linear fit algorithms, were applied over the variables, and mean-centered processing was applied over the samples, which reduced the noise and minor analytical errors [38,39]. For HCA, the matrix was mean-centered, and the incremental linkage method (sum-of-squares approach in calculating the inter-cluster distances) was applied. The Euclidian distance was used for distance metric. The PCA was performed using singular value decomposition (SVD) algorithm. To improve the identification of the chemical constituents associated with cashew genotype, a supervised method by PLS-DA was employed using the SIMPLS algorithm. The number of LVs were selected in accordance with the statistical parameters: RMSEC; RMSECV; calibration and cross-validation coefficients (r^2^); and similarity criterion RMSEC/RMSECV ratio above 0.75 [16,21].

#### 4.3.2. Relative Contribution

The peaks detected as exactly as possible, in both m/z and retention time, were used for determining the peak area for achieving the relative contribution of the compounds with less overlapped signals in the chromatograms. The relative contribution of the areas was calculated based on the total ion abundance from the peaks in the samples, since the relative amplitude of the peaks provides the relative abundance of the isotopic forms in the chromatograms. Therefore, the normalized means in the base peak intensity (BPI) at the retention times of 1.60, 2.82, 3.28, 3.83, and 4.25 min were determined.

The results were evaluated using the analysis of variance ANOVA single factor (significance level of 0.05; means comparison using Tukey test; Levene’s test for the homogeneity of the variance) to statistically certify the differences among the relative contributions. The deviation of the method was estimated based on the null hypothesis (*p*-value) from the three replicates of sampling for two years, totaling 6 samples for each cashew genotype.

## 5. Conclusions

It was demonstrated that MicroNIR spectroscopic analyses of the cashew apple composition provided a non-destructive and low-cost method for obtaining prompt results. In addition, important composition tendencies were observed with four fruits clusterings according to their composition similarity, and genotype, even considering the morphologic differences, including shape, size, and color. The multivariate regression results, obtained using °Brix and total acidity, showed that it is possible to satisfactorily predict °Brix and total acidity within the cashew genotypes. However, the parameters related to the fruit composition, and the environmental factors that affect the instrument performance must be taken into account to improve the comprehension of the chemical and physical variability of the cashew apples from the germplasm bank of Embrapa.

Additionally, the chemometrics evaluation of the UPLC-HRMS dataset was suitable to follow changes in the composition of cashew apple pulps according to genotype. The current study resulted in the identification of relatively high amounts of different bioactive compounds, including galloylhexose I, digalloylhexoside I, hydroxybutanoic acid ethyl ester-hexoside, and the flavonoids, myricetin-3-*O*-glucoside and myricetin-3-*O*-rhamnoside, in different genotypes. This information is useful for breeding programs to establish accessions with higher concentrations of important compounds for human health.

## Figures and Tables

**Table 1:** Statistical parameters obtained by multivariate regression modeling of MicroNIR spectra with °Brix and total acidity using 3 LV.

Model	3 LV^1^ (%)	r^2^ cal^2^	RMSEC^3^	r^2^ CV^4^	RMSECV^5^	RMSEC / RMSECV^6^	Bias^7^	CV Bias^8^
°Brix	96.5	0.74	0.11	0.46	0.16	0.69	3.3 × 10^−15^	−0.004
Acidity	97.1	0.66	1.19	0.46	1.53	0.78	1.8 × 10^−15^	0.064

^1^ Percentage variance captured by the regression model;

^2^ Coefficient of correlation between the real and predicted values during the calibration;

^3^ Root mean square error of calibration;

^4^ Coefficient of correlation between the real and predicted values during the cross-validation;

^5^ Root mean square error of cross-validation;

^6^ Similarity criterion;

^7^ Average difference between the estimator and real values during the calibration;

^8^ Average difference between the estimator and real values during the cross-calibration.

**Table 2:** Parameters from partial least squares-discriminant analysis (PLS-DA) classification model of UPLC-HRMS data from cashew apple pulps of different genotypes.

Model	LV1+LV2+LV3^1^	r^2^ cal^2^	RMSEC^3^	r^2^ val^4^	RMSECV^5^	RMSEC / RMSECV^6^
PLS-DA	88.05%	0.88	0.298	0.85	0.341	0.874

^1^ Total variance percent in X matrix refer to 3 LVs; ^2^ Coefficient of correlation between the real and predicted groups during the calibration; ^3^ Root mean square error of calibration; ^4^ Coefficient of correlation between the real and predicted groups during the cross-validation; ^5^ Root mean square error of cross-validation; ^6^ Similarity criterion.

**Table 3:** Illustration of the cashew genotypes associated with the accession number and morphoagronomic characteristics: plant size, tree appearance, fruits color and shape, and origin (county-state).

Accession Number	Plant Size	Tree Appearance	Fruit Color	Fruit Shape	Sampling Origin	Illustration
CP 76	tall	Open erect	orange	pyriform	crop* / Maranguape-CE	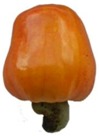
Clone 98/101	semi tall	compact erect	orange	pyriform	breeding program* / Pacajus-CE	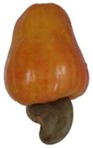
Progeny 2005/127	semi tall	compact erect	dark red	pyriform	breeding program/ Beberibe-CE	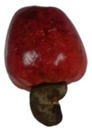
Progeny 2005/133	semi tall	open erect	orange	spherical	breeding program/ Cruz-CE	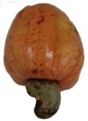
BRS 226	dwarf	compact erect	orange	pyriform	crop / Pio IX-PI	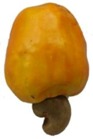
Clone 2005/102	tall	compact erect	orange	pyriform	breeding program/ Beberibe-CE	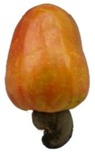
CP 09	semi tall	compact erect	orange	pyriform	crop / Maranguape-CE	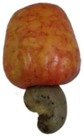
B 393	tall	compact erect	light red	spherical	germplasm* / Aracati- CE	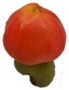
BRS 275	semi tall	open erect	orange	pyriform	crop / Pacajus-CE and Maranguape-CE	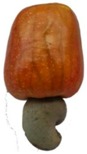
B 963	tall	open erect	yellow orange	pyriform	germplasm / Maranguape-CE	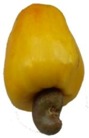
Hybrid 2001/3	semi tall	compact erect	orange	pyriform	breeding program/ Maranguape-CE and Pio IX–PI	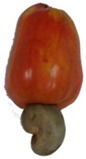
Hybrid 2001/6	semi tall	compact erect	yellow orange	pyriform	breeding program/ Maranguape-CE and Pio IX-PI	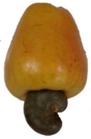
B 967	tall	open erect	orange	cylindrical	germplasm / Pacajus-CE	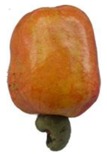
CP 06	tall	open erect	yellow	conical obovate	crop / Pacajus-CE	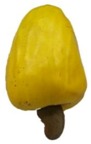
Progeny 2005/122	semi tall	open erect	yellow	spherical	breeding program/ Beberibe-CE	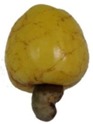
Hybrid 2001/13	semi tall	open erect	orange	pyriform	breeding program/ Pacajus-CE	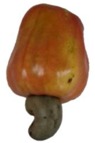
Clone 2005/111	semi tall	open erect	orange	pyriform	breeding program/ Serra do Mel-RN	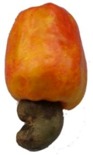
Clone 98/116	semi tall	open erect	orange	pyriform	breeding program/ São Luiz do Curu-CE	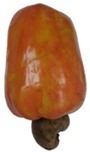
B 741	semi tall	compact erect	orange	pyriform	breeding program (CP 76 x *A. microcarpum*) / Maranguape-CE	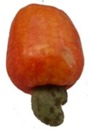
Progeny 2005/223	semi tall	open erect	orange	pyriform	breeding program/ Beberibe-CE	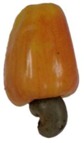
Embrapa 51	semi tall	open erect	orange	pyriform	crop / Pacajus-CE	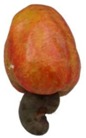
M 886	tall	open erect	yellow	spherical	breeding program/ Beberibe-CE	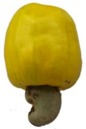
1001	tall	open erect	orange	pyriform	crop/ Pacajus-CE	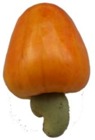
Clone 2003/102	semi tall	compact erect	orange	pyriform	breeding program/ Pio IX -PI	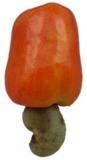

* Legends: crop means registered product on market; breeding program means plant before crossbreed; germplasm means plant collected and conserved in the germplasm bank.

## Supplementary Materials

The following are available online at https://www.mdpi.com/2218-1989/9/6/121/s1. Figure S1: (a) Raw absorbance spectra from 135 cashew apples obtained using the portable NIR spectrometer, and the same spectra after the following treatments, (b) MSC, (c) SNV, and (d) first derivative using the Savitzky–Golay filter with a second order polynomial for five points. Figure S2: The total ion chromatograms from 24 different genotypes of cashew apple pulps acquired under negative ionization mode. Figure S3: Dendrogram representing the chemical composition similarity relationships among the cashew apple pulps. Figure S4: Regression modeling using the MicroNIR dataset from different genotypes of cashew apples based on °Brix values: a) influence plot of Hotelling’s T^2^ × Q residuals, (b) leverage × studentized residuals, c) Y calibration × cross-validated Y with 95% confidence limits, and d) scores on LV1 × LV2. Figure S5: Regression modeling using the MicroNIR dataset from different genotypes of cashew apples based on total acidity: a) influence plot of Hotelling’s T^2^ × Q residuals, (b) leverage × studentized residuals, c) Y calibration × cross-validated Y with 95% confidence limits, and d) scores on LV1 × LV2.
