# Supplementary material for: Metabolomic Variability of Different Genotypes of Cashew by LC-Ms and Correlation with Near-Infrared Spectroscopy as a Tool for Fast Phenotyping
Source: Metabolites. 2019 Jun 25;9(6):121. doi: 10.3390/metabo9060121 (PMC6630256; doi:10.3390/metabo9060121)
Supplement: Supplementary file 1 [file metabolites-09-00121-s001.pdf]

Metabolomic variability of different genotypes of cashew by LC-MS and correlation with near-infrared spectroscopy as a tool for fast phenotyping

Elenilson Alves Filho [1], Lorena Mara Silva [2], Ynayara Lima [3], Paulo Ribeiro [2], Ebenézer Silva [2], Guilherme Zocolo [2], Kirley Canuto [2], Selene Morais [3], Ana Cecília Castro [2] and Edy de Brito [2,*]

[1] Department of Food Engineering, Universidade Federal do Ceará, Fortaleza-CE, 60020-181 Brazil; elenilson.godoy@yahoo.com.br

[2] Embrapa Agroindústria Tropical, Rua Dra Sara Mesquita, 2270, Pici, 60511-110 Fortaleza-CE, Brazil; lorena.mara@embrapa.br (L.M.S.); paulo.riceli@embrapa.br (P.R.); ebenezer.silva@embrapa.br (E.S.); guilherme.zocolo@embrapa.br (G.Z.); kirley.canuto@embrapa.br (K.C.); cecilia.castro@embrapa.br (A.C.S.)

[3] Centro de Ciências e Tecnologia, Universidade Estadual do Ceará, Fortaleza-CE, 60020-181 Brazil; selenemaiademorais@gmail.com

* Correspondence: edy.brito@embrapa.br; Tel.: +55-082-33917393

1. Pretreatment of the numerical matrices for chemometric analysis

Figure 1SI illustrates the different pretreatments tested to reach the information quality for chemometric analysis.

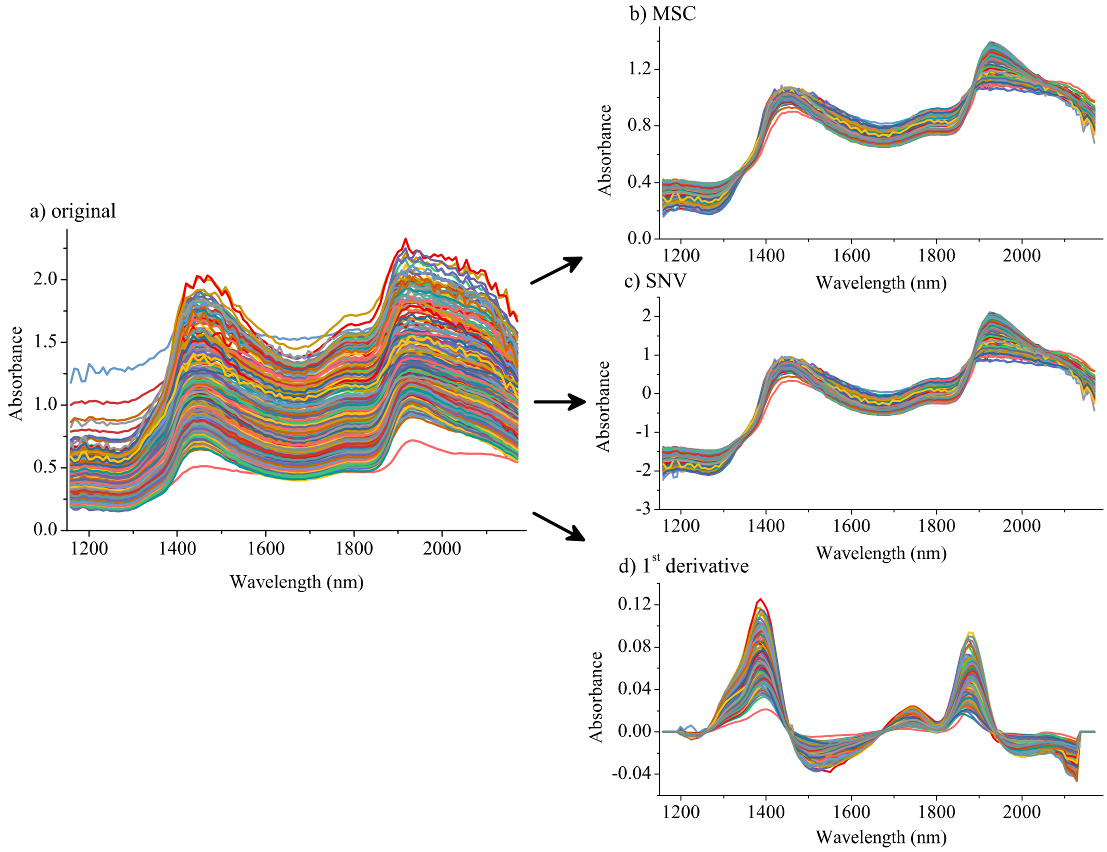

Figure S1. (a) Raw absorbance spectra from 135 cashew apple obtained using the portable NIR spectrometer, and the same spectra after the follow treatment; (b) MSC; c) SNV; and d) first derivative using Savitzky-Golay filter with a second order polynomial for five points.

All the chromatogram peaks presented at Figure 2SI were previously aligned using the algorithm COW (Correlation Optimized Warping).

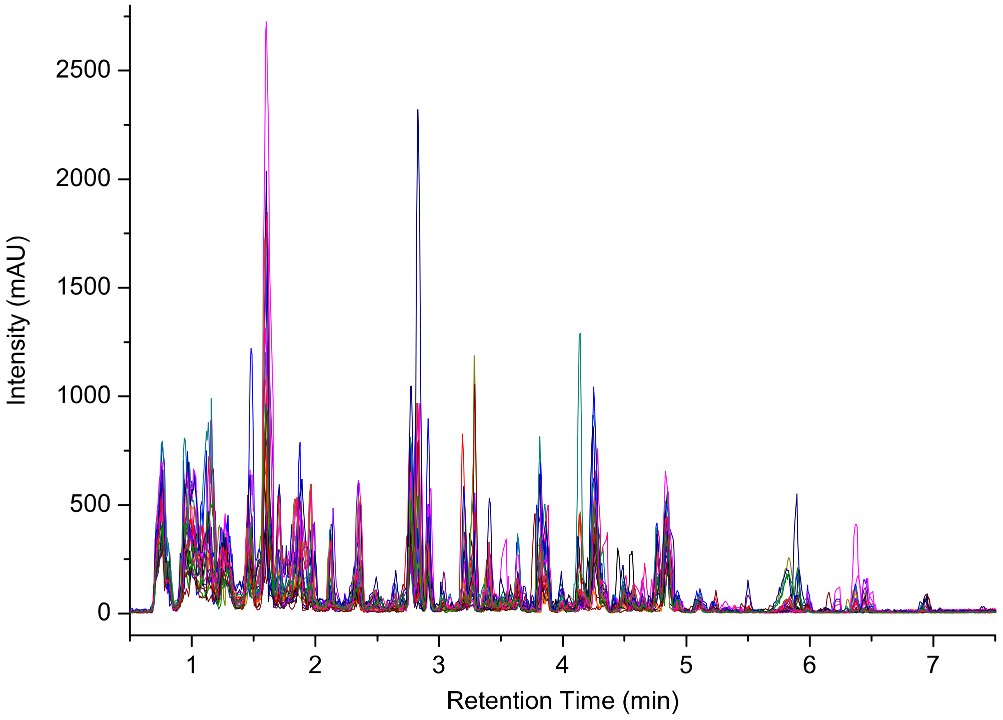

Figure S2. Total ion chromatograms from 24 different genotypes of cashew apple pulps acquired under negative ionization mode.

The unsupervised method based on HCA was applied to segregate the samples in groups regarding the composition similarity, which is presented in Figure 3SI.

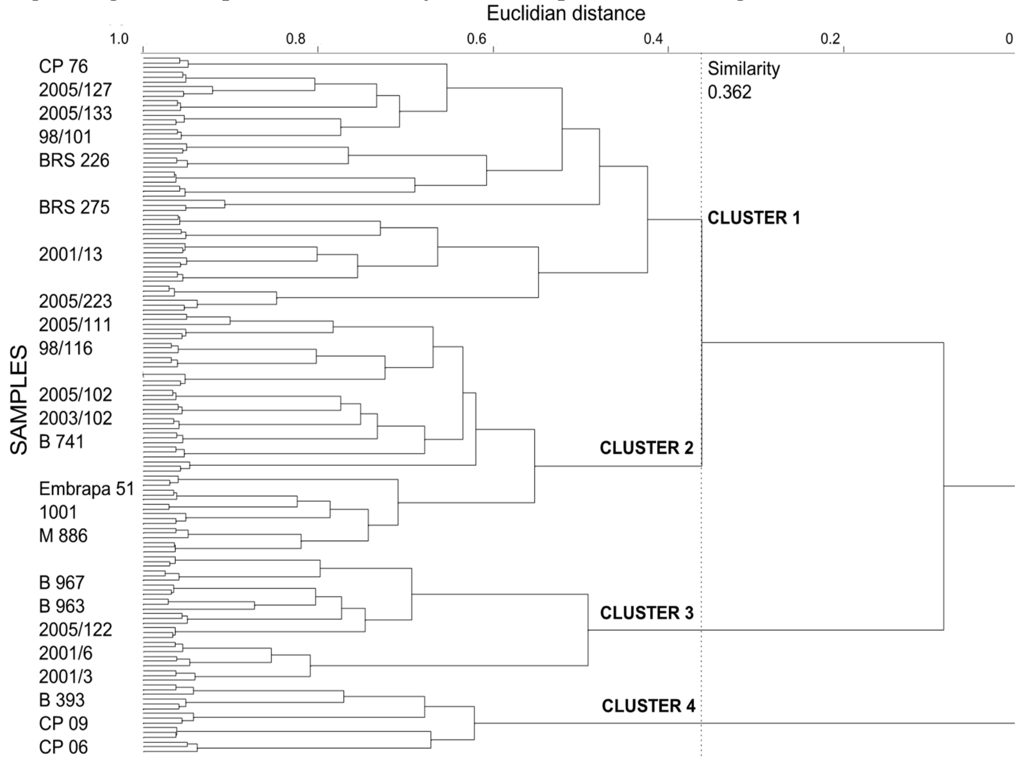

Figure S3. Dendrogram representing the chemical composition similarity relationships among the cashew apple pulps.

Table S1 – Biomarkers* present in cashew apple pulps by UPLC-QTOF-MSE

Peak n°.	t_R (min)	Theoretical [M−H]− ions (m/z)	Calculated ion formulas [M−H]−	Error (ppm)	Major fragment ions	Calculated molecular formulas	Compound	Reference
1	1.6	331.0665	331.0650	-4.5	169.0118; 125.0231	$C_{13}H_{16}O_{10}$	Galloylhexose I	[1]
2	2.82	483.0775	483.0741	-7.0	331.0691; 169.0141; 125.0236	$C_{20}H_{20}O_{14}$	Digalloyl-hexoside I	[1]
3	3.28	293.1231	293.1236	1.7	131.0699 113.0269	$C_{12}H_{21}O_8$	hydroxybutanoic acid ethyl ester-hexoside	[2]
4	3.83	479.0812	479.0826	-2.9	317.0250 316.0215 169.0227	$C_{21}H_{20}O_{13}$	Myricetin-3-Oglucoside	[1,3]
5	4.25	463.0877	463.0875	-0.4	317.0285 316.0228	$C_{21}H_{20}O_{12}$	Myricetin 3-O-rhamnoside	[4,5]

* The identification was performed by using the retention time, MS and MS/MS spectra of each cashew apple pulps, along with the fragmentation mechanism, reference data related to family (Anacardiaceae), genus (*Anacardium*), and species (*occidentale*), and the SciFinder, ChemSpider, PubChem databases, and literature reports.

Figures 4S and 5S presents the marit graphs from the regression modeling using °Brix and total acidities, respectively. The higher concentrations are represented by dark red, and the lower in dark blue. Figures 3a and 4a illustrate the influence biplot with cashew apple distribution according to Hotelling T^2 × Q residuals, which present the absence of outliers (both values above 1, upper left quadrant) [6]. Some cashew apples presented high influence on regression model (highest T^2) and high residuals (error) for modeling. According to Figures 3b and 4b, the leverage plot reveled the influence of each genotype on models based on Hotelling T^2, and the studentized residuals (mean zero and unit variance) indicated the lack of fit of some quantitative parameters [7]. However, despite the high leverage and high residuals, the respective samples expressed a very low studentized Y residual and, therefore, the regression model was still able to sufficiently predict °Brix and total acidity based on selected MicroNIR spectra (green region – Fig. 2). Figures 3c (°Brix) and 4c (total acidity) describe the robustness of the prediction model by the proximity between both regression curves from calibration and crow validation (red and green lines). Figures 3d and 4d illustrate the new samples positioning regarding the amounts of °Brix and total acidity, since the axes lose their orthogonality to adjust to the variations of the dependent variables [7].

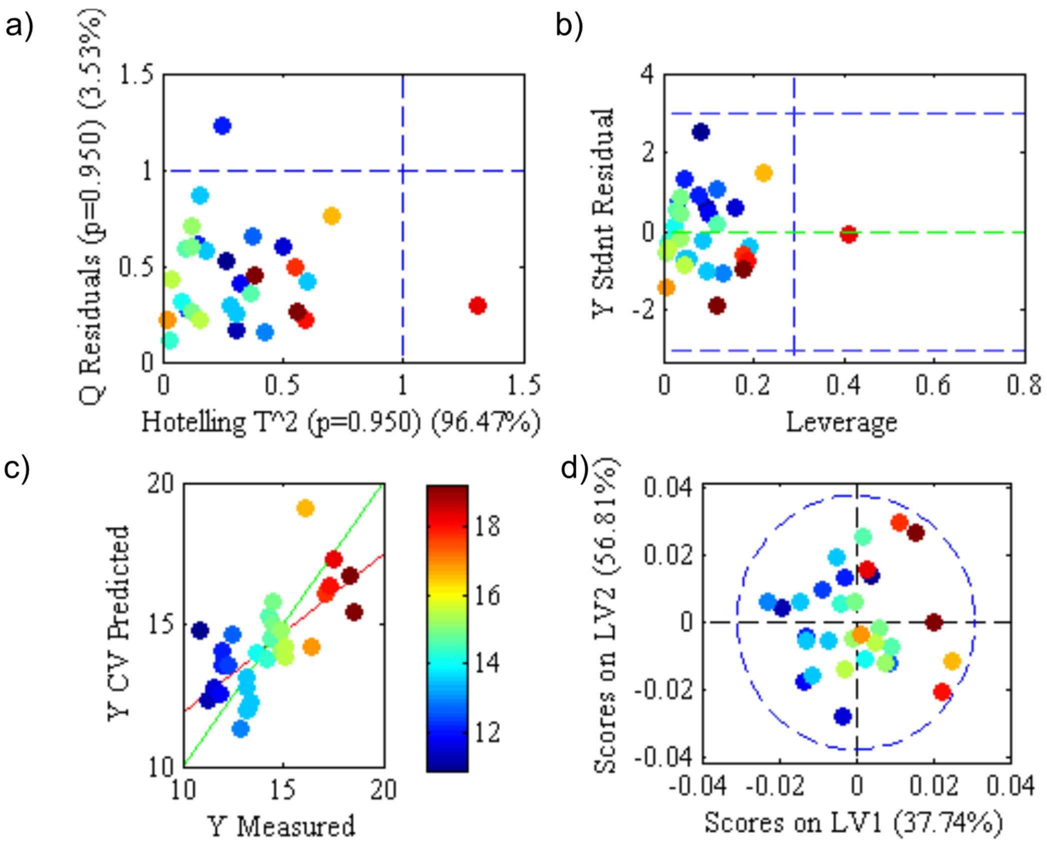

Figure S4. Regression modeling using the MicroNIR dataset from different genotypes of cashew apples based on °Brix values: a) influence plot of Hotelling T^2 × Q residuals; b) leverage × studentized residuals; c) Y calibration × cross-validated Y with 95% confidence limits; d) scores on LV1 × LV2.

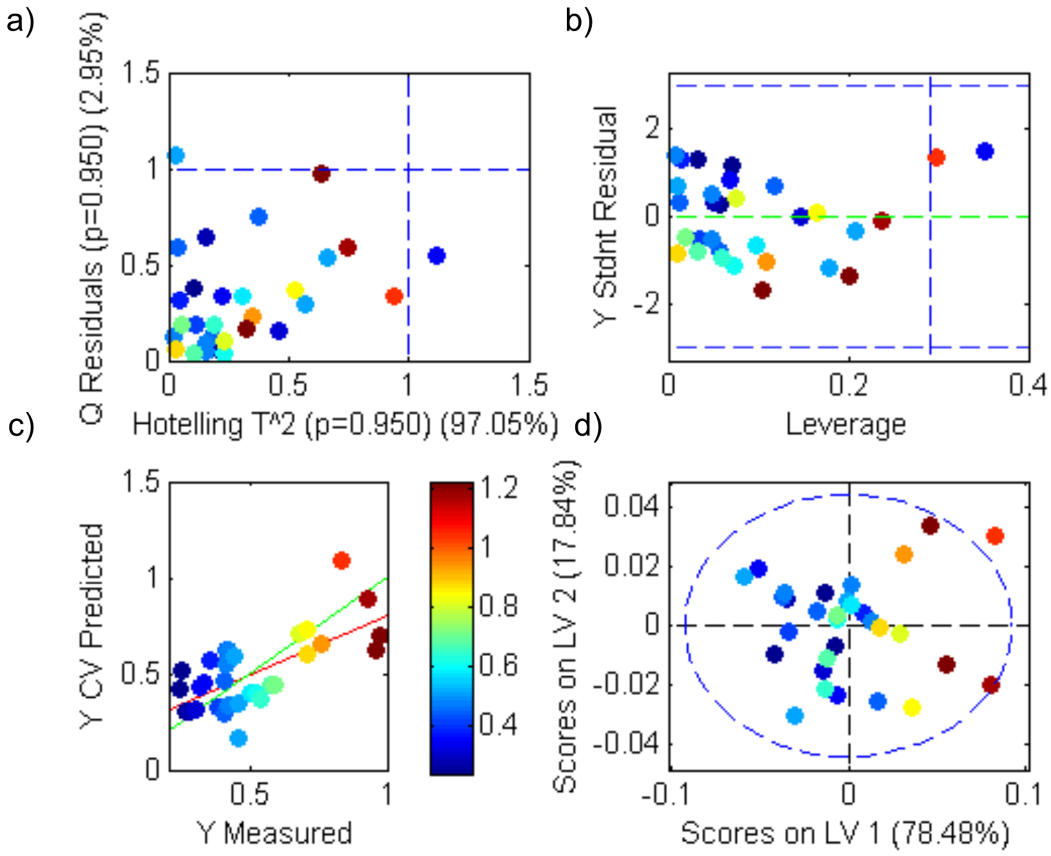

Figure S5. Regression modeling using the MicroNIR dataset from different genotypes of cashew apples based on total acidity: a) influence plot of Hotelling $T^2 \times$ Q residuals; b) leverage × studentized residuals; c) Y calibration × cross-validated Y with 95% confidence limits; d) scores on LV1 × LV2.

References

1. Abu-Reidah, I.M.; Ali-Shtayeh, M.S.; Jamous, R.M.; Arráez-Román, D.; Segura-Carretero, A. HPLC–DAD–ESI-MS/MS screening of bioactive components from Rhus coriaria L. (Sumac) fruits. *Food Chemistry* **2015**, *166*, 179-191, doi:10.1016/j.foodchem.2014.06.011.
2. Rodríguez-Pérez, C.; Quirantes-Piné, R.; Fernández-Gutiérrez, A.; Segura-Carretero, A. Comparative characterization of phenolic and other polar compounds in Spanish melon cultivars by using high-performance liquid chromatography coupled to electrospray ionization quadrupole-time of flight mass spectrometry. *Food Research International* **2013**, *54*, 1519-1527, doi:10.1016/j.foodres.2013.09.011.
3. Regazzoni, L.; Arlandini, E.; Garzon, D.; Santagati, N.A.; Beretta, G.; Maffei Facino, R. A rapid profiling of gallotannins and flavonoids of the aqueous extract of Rhus coriaria L. by flow injection analysis with high-resolution mass spectrometry assisted with database searching. *Journal of Pharmaceutical and Biomedical Analysis* **2013**, *72*, 202-207, doi:10.1016/j.jpba.2012.08.017.
4. Shukri, M.A.M.; Alan, C. Analysis of phenolics in Anacardium occidentale shoot extracts using a reversed-phase high performance liquid chromatography tandem mass spectrometry (RP-HPLC-MS). *Journal of Tropical Agriculture and Food Science* **2010**, *38*, 221-230.
5. Dorta, E.; González, M.; Lobo, M.G.; Sánchez-Moreno, C.; de Ancos, B. Screening of phenolic compounds in by-product extracts from mangoes (Mangifera indica L.) by

HPLC-ESI-QTOF-MS and multivariate analysis for use as a food ingredient. *Food Research International* **2014**, *57*, 51-60, doi:10.1016/j.foodres.2014.01.012.

6. Ballabio, D. A MATLAB toolbox for Principal Component Analysis and unsupervised exploration of data structure. *Chemometrics and Intelligent Laboratory Systems* **2015**, *149*, 1-9, doi:10.1016/j.chemolab.2015.10.003.
7. Wise, B.M.; Gallagher, N.; Bro, R.; Shaver, J.; Windig, W.; Koch, R.S. PLS Toolbox 4.0. *Eigenvector Research Incorporated: Wenatchee, WA, USA* **2007**.
